# Therapeutic potential of extracellular vesicle‐associated long noncoding RNA

**DOI:** 10.1002/btm2.10172

**Published:** 2020-07-16

**Authors:** Louis J. Born, John W. Harmon, Steven M. Jay

**Affiliations:** ^1^ Fischell Department of Bioengineering University of Maryland College Park Maryland USA; ^2^ Department of Surgery and Hendrix Burn/Wound Laboratory Johns Hopkins University School of Medicine Baltimore Maryland USA; ^3^ Program in Molecular and Cell Biology University of Maryland College Park Maryland USA

**Keywords:** exosomes, extracellular vesicles, lncRNA, microparticles, microvesicles

## Abstract

Both extracellular vesicles (EVs) and long noncoding RNAs (lncRNAs) have been increasingly investigated as biomarkers, pathophysiological mediators, and potential therapeutics. While these two entities have often been studied separately, there are increasing reports of EV‐associated lncRNA activity in processes such as oncogenesis as well as tissue repair and regeneration. Given the powerful nature and emerging translational impact of other noncoding RNAs such as microRNA (miRNA) and small interfering RNA, lncRNA therapeutics may represent a new frontier. While EVs are natural vehicles that transport and protect lncRNAs physiologically, they can also be engineered for enhanced cargo loading and therapeutic properties. In this review, we will summarize the activity of lncRNAs relevant to both tissue repair and cancer treatment and discuss the role of EVs in enabling the potential of lncRNA therapeutics.

**Abbreviations**
CRCs: colorectal carcinoma cells
EVs: extracellular vesicles
hAD‐MSCs: human adipose‐derived mesenchymal stem cells
HCAECs: human coronary artery endothelial cells
HCC: hepatocellular carcinoma cell
HDFs: human dermal fibroblasts
HDMECs: human dermal microvascular endothelial cells
lncRNA: long noncoding RNA
miRNA: microRNA
SCCs: squamous carcinoma cells

## INTRODUCTION

Extracellular vesicles (EVs), including exosomes, microvesicles, and other subtypes, have emerged as a novel class of cell‐derived therapeutics with vast potential. EVs are released from virtually every cell type and are capable of transferring lipids, proteins, and nucleic acids to recipient cells in paracrine or endocrine fashion.^1^, ^2^ A majority of studies to date have specified proteins and/or microRNAs (miRNAs) as the primary therapeutic components of EVs; however, long noncoding RNAs (lncRNAs) are increasingly being recognized as important mediators of EV biological effects. lncRNAs are defined as any RNA over 200 bps with no apparent coding function,^3^, ^4^, ^5^ and their abilities to interact with cellular miRNAs via both complementary sequence binding and secondary structure effects have been linked to therapeutic outcomes.^6^, ^7^ Intercellular transfer of lncRNAs is naturally accomplished by EVs, and thus there is particular interest in and potential for harnessing this mechanism for therapeutic benefit. This review summarizes research on the therapeutic activity of lncRNA and EV‐associated lncRNA to date relevant to both tissue repair and cancer treatment. It further discusses current knowledge and expected challenges of EV‐mediated lncRNA delivery as well as issues for future consideration to enable translation of EV‐associated lncRNA therapeutics.

## THERAPEUTIC ACTIVITY OF lncRNA FOR TISSUE REPAIR AND REGENERATION

lncRNAs play diverse regulatory roles in normal physiological processes. Thus, delivery of lncRNAs via EVs may help in the treatment of diseases and injuries across all organ systems. In this section, therapeutic effects of lncRNAs relevant to the integumentary, musculoskeletal, cardiovascular, nervous, and gastrointestinal systems are discussed.

**TABLE 1:** lncRNA activity in the integumentary system

lncRNA	Bioactivity	Reference
H19	Promotes endothelial and fibroblast activity	^9^, ^14^, ^28^
MALAT1	Promotes endothelial and fibroblast activity	^10^, ^11^, ^29^
HOTAIR	Promotes endothelial activity	^11^
XIST	Promotes fibroblast activity	^15^
WAKMAR1	Promotes keratinocyte activity	^17^
WAKMAR2	Promotes keratinocyte activity	^18^
GAS5	Promotes keratinocyte activity	^19^
PlncRNA‐1	Promotes hair follicle activity	^23^
lncRNA5322	Promotes hair follicle activity	^24^

Abbreviation: lncRNA, long noncoding RNA.

**TABLE 2:** lncRNA activity in the musculoskeletal system

lncRNA	Bioactivity	Reference
KLF3‐AS1	Promotes chondrocyte activity	^32^, ^33^
PMS2L2	Promotes chondrocyte activity	^34^
MALAT1	Reduces inflammatory injury of chondrocytes	^35^
NEAT1	Promotes osteogenic differentiation	^39^
GAS5	Promotes apoptosis of fibroblast‐like synoviocytes	^40^, ^41^
HOTAIR	Promotes chondrocyte activity and inhibit inflammation	^42^
MALAT1	Promotes endothelial activity	^43^
OG	Promotes osteogenic differentiation	^44^

Abbreviation: lncRNA, long noncoding RNA.

**TABLE 3:** lncRNA activity in the cardiovascular system

lncRNA	Bioactivity	Reference
H19	Upregulates VEGFA in myocardial infarction model	^46^
VEGF‐AS1	Upregulates VEGFA during hypoxia	^47^
VEGF‐AS2	Upregulates VEGFA during hypoxia	^47^
UCA1	Increases cardioprotective protein HSP70	^48^
GAS5	Decreases cardiomyocyte apoptosis by inhibiting Sema3a	^49^
CCRR	Improves cardiac conduction by preventing degradation of connexin43	^50^
MALAT1	Inhibits NF‐κB/TNF‐α pathway in cardiac inflammation and regulate cardioprotective effects of myricetin	^51^, ^52^

Abbreviation: lncRNA, long noncoding RNA.

**TABLE 4:** lncRNA activity in the nervous system

lncRNA	Bioactivity	Reference
MALAT1	Protects blood brain barrier and enhances neuronal survival in ischemic stroke model. Aids in recovery in traumatic brain injury model.	^53^, ^54^, ^56^
H19	Protects against oxygen and glucose deprivation in PC12 cells	^55^

Abbreviation: lncRNA, long noncoding RNA.

**TABLE 5:** lncRNA activity in the gastrointestinal system

lncRNA	Bioactivity	Reference
H19	Reduces liver damage in acute liver failure model, and promotes activity of intestinal cells in inflammatory injured intestine	^57^, ^59^
Gas5	Restrains hepatic fibrosis in hepatic fibrosis model	^58^

Abbreviation: lncRNA, long noncoding RNA.

### Integumentary system

The integumentary system consists of the skin, hair, and nails, as well as exocrine glands, and is responsible for providing protection from the environment. Wound healing is an essential process in dealing with insults that breach this protective barrier. Endothelial cells play a major role in wound healing, as the formation of new blood vessels is crucial for tissue repair and regeneration.^8^ Increasing the proliferative and migratory properties of these cells is required for new blood vessels to form and facilitate healing. Aimed at this goal, Tao et al. utilized extracellular mimics to deliver lncRNA H19, resulting in improved proliferation, migration, and tube formation of human dermal microvascular endothelial cells. Further, using a streptozocin‐induced diabetic mouse model, they showed that delivery of H19‐containing vesicles improved blood supply to the wound and helped aid healing overall. The angiogenic effects observed were attributed to potential H19 involvement in the insulin–PI3K–Akt pathway.^9^

**FIGURE 1:**
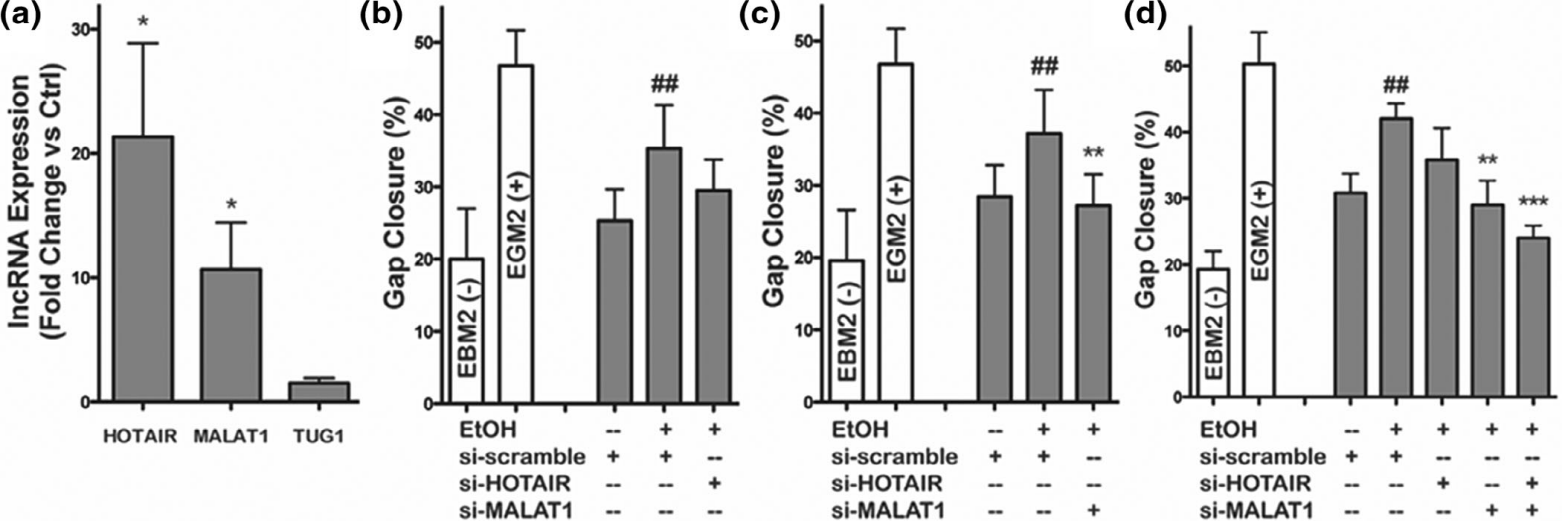
Role of long noncoding RNA (lncRNA) in endothelial cell (EV)‐derived bioactivity. (a) Expression levels of the indicated lncRNAs were assessed by qPCR in EVs from human umbilical vein endothelial cells (HUVECs) cultured in the presence versus absence of 100 mM ethanol (EtOH) for 24 hr (*n* = 3; *p < .05). (b–d) HUVEC gap closure was assessed following 24 hr stimulation by 100 μg/ml EVs from HUVECs cultured in the absence (−EtOH) or presence (+EtOH) of 100 mM EtOH for 24 hr following transfection with a scrambled small interfering RNA (siRNA) (scr) or siRNA specific to (b) HOTAIR, (c) MALAT1, or (d) both HOTAIR and MALAT1 (double transfection) (*n* = 4; ##*p* < .01 vs. − EtOH + scr; **p < .01, ***p < .001 vs. + EtOH + scr). HUVECs incubated in basal medium (EBM2, without growth factors) were used as the negative control (−) and HUVECs incubated in growth medium (EGM2, with growth factors) were used as positive controls (+). Data reproduced from Reference 11 (open access) with no changes

Endothelial cells are also a potential source for EVs with therapeutic lncRNAs. Shyu et al. altered the environmental conditions of human coronary artery endothelial cells to augment levels of lncRNA MALAT1 in their secreted exosomes. They delivered these exosomes to ischemic hindlimbs of rats and saw improved neovascularization via a potential mechanism of action of sequestration of miR‐92a and upregulation of KLF2.^10^ In a separate study, Lamichhane et al. altered environmental conditions of human umbilical vein endothelial cells (HUVECs) by supplementing media with low doses of ethanol. EVs collected from these preconditioned cells were found to have increased levels of lncRNAs HOTAIR and MALAT1 that produced enhanced vascularization responses in vitro and in vivo (Figure 1).^11^ This phenomenon was replicated in a scalable bioreactor culture system, demonstrating the potential to produce EVs with specifically enhanced therapeutic lncRNA content at large scale.^12^

In addition to endothelial cells, fibroblasts are also crucial players in wound healing as they deposit extracellular matrix in newly formed skin and release a variety of growth factors that help orchestrate the angiogenic component of wound repair.^13^ Copper et al. assessed the effects of exosomes from human adipose‐derived mesenchymal stem cells (hAD‐MSCs) on human dermal fibroblasts (HDFs). Unmodified hAD‐MSC exosomes increased HDF migration in vitro, but this effect was reduced when the hAD‐MSCs were transfected with an antisense oligonucleotide to MALAT1 prior to exosome collection. When hAD‐MSC‐conditioned media was applied to an ischemic excisional wound healing model, wound closure improved compared to control media treatment.^14^ Additionally, a study employing a skin fibroblast in vitro thermal injury model demonstrated that fibroblast proliferation, migration, and extracellular matrix synthesis was enhanced through the upregulation of lncRNA XIST by downregulating miR‐29a and upregulating Lin28a.^15^

Full keratinocyte coverage (i.e., epithelization) is the defining clinical criteria of a closed wound.^16^ Li et al. identified novel roles for two lncRNAs—LOC105372576 (renamed WAKMAR1 [wound and keratinocyte migration‐associated lncRNA 1]),^17^ and LOC100130476 (renamed (WAKMAR2 [wound and keratinocyte migration‐associated lncRNA 2])^18^—that both increase keratinocyte migration in wound beds. Another study by Sawaya et al. found that mevastatin upregulated lncRNA GAS5, which led to the inhibition of c‐myc, a transcription factor that inhibits keratinocyte migration and whose overexpression has also been shown to be a hallmark of chronic wounds.^19^

Beyond the cell types mentioned above, hair follicles contain a number of stem cells at their base that play a role in wound healing.^20^, ^21^, ^22^ Si et al. found that transfecting hair follicle stem cells with the lncRNA PlncRNA‐1 increased their proliferation and differentiation. Treatment of these transfected cells with the TGF‐β1 inhibitor LY2109761 decreased these effects, suggesting a role for PlncRNA‐1 in the TGF‐β1‐mediated Wnt/β‐catenin signaling cascade.^23^ In a similar vein, Cai et al. found that transfection of hair follicle stem cells with IncRNA5322 also promoted their proliferation and differentiation due to the activation of a miR‐21‐mediated PI3K‐AKT signaling pathway.^24^

Finally, transfusions of autologous blood and blood‐derived products have been developed to address chronic wounds.^25^, ^26^, ^27^ Guo et al. utilized autologous blood transfusions in a streptozocin‐induced diabetic mouse model in which lncRNA H19 was upregulated, improving wound healing. Mechanistically, they showed that H19 increased the expression of the angiogenic protein HIF‐1α in fibroblasts by methylating histones via H3K4me3.^28^ Another transfusion study by Liu et al. investigated the effects of autologous blood transfusions on wound healing in a similar diabetic mouse model. Mice treated with autologous blood efficacious in speeding wound healing showed increased levels of lncRNA MALAT1, which was found to activate fibroblasts through the initiation of the HIF‐1α signaling pathway.^29^ Key data are summarized in Table 1.

### Musculoskeletal system

Osteoarthritis is the most common joint disorder in western populations and is characterized by chronic degradation of articular cartilage as well as osteophyte formation.^30^, ^31^ With regard to cartilage, Liu et al. showed that exosomes derived from human MSCs contained lncRNA KLF3‐AS1and promoted chondrocyte proliferation in vitro. In an in vivo collagenase‐induced model of osteoarthritis, the same MSC exosomes improved cartilage repair.^32^ The same group explored the molecular action of KLF3‐AS1 and found that it sponges miR‐206 leading to the upregulation of G‐protein‐coupled receptor kinase interacting protein‐1 and resulting in chondrocyte proliferation and apoptosis inhibition.^33^ A different study by Li et al. found that the lncRNA PMS2L2 served a protective role in an in vitro LPS‐induced osteoarthritis model. The lncRNA upregulated myeloid cell leukemia‐1 by sponging miR‐203, causing increased chondrocyte viability and decreased apoptosis.^34^ Interestingly, in a study by Pan et al., lncRNA MALAT1 was able to upregulate miR‐19b, contributing to the inactivation of Wnt/β‐catenin and NF‐κB pathways. This ultimately led to a reduction of LPS‐induced inflammatory injury in murine ATDC5 cells.^35^

Osteoporosis is characterized by skeletal fragility and microarchitectural deterioration,^36^ and differentiating bone marrow stromal cells to osteoblasts as a treatment has been an active area of research.^37^, ^38^ Zhang et al. showed that osteogenic differentiation of bone marrow‐derived MSCs was promoted by lncRNA NEAT1 via sponging miR29b‐3p to upregulate BMP1.^39^ Additionally, Wang et al. utilized microgravity effects on the osteoblast precursor cell line MC3T3‐E1 as well as in an in vivo hind limb unloading model for bone loss to demonstrate that lncRNA ODSM sponged miR‐139‐3p to upregulate ELK1.^40^

Rheumatoid arthritis is characterized by invasive fibroblast‐like synoviocytes that cause joint destruction.^36^ Li et al. showed that the phytochemical Tanshinone IIA was able to promote the apoptosis of these fibroblast‐like synoviocytes by upregulating lncRNA GAS5.^41^ Another study by Zhang et al. utilized LPS‐induced chondrocytes and showed that lncRNA HOTAIR inactivated NF‐κB signaling by downregulating miR‐138. In rheumatoid arthritis animal models, it was found that chondrocyte proliferation was upregulated and inflammatory markers IL‐17 AND IL‐23 were downregulated by HOTAIR.^42^

Bone fractures primarily heal by formation of a callus, which is eventually vascularized and calcified. Ciu et al. showed that endothelial progenitor cells cocultured with bone marrow‐derived macrophages released exosomes enriched with lncRNA MALAT1. MALAT1 was found to sequester miR‐124 and subsequently upregulate integrin subunit β1 as well as promote neovascularization at the bone fracture site in an in vivo mouse bone fracture model. Healing was improved in mice treated with these MALAT1‐containing exosomes compared to those administered from bone marrow‐derived macrophages.^43^ In addition, Tang et al. found that lncRNA OG interacts with heterogeneous nuclear ribonucleoprotein K to regulate the expression of BMP family proteins to promote osteogenic differentiation of bone marrow‐derived MSCs.^44^ Key data are summarized in Table 2.

### Cardiovascular system

Common cardiovascular ailments include ischemic heart disease, ischemia–reperfusion injury, arrhythmias, and inflammation, among others.^45^ Related to ischemic heart disease, Hou et al. found that overexpressing lncRNA H19 improved the therapeutic benefit of MSC transplantation to regions of local myocardial infarct by sequestering miR199a‐5p and consequently upregulating vascular endothelial growth factor A (VEGF‐A).^46^ Also, Nieminen et al. found that two antisense lncRNAs—RP1‐261G23.7 (VEGF‐AS1) and EST AV731492 (VEGF‐AS2)—interact at the VEGF‐A promotor and are concordantly upregulated with VEGF‐A during hypoxia.^47^ Studying ischemia–reperfusion injury, a possible sequela of myocardial infarction, Chen et al. found that the upregulation of lncRNA UCA1 contributed to the benefits seen from morphine postconditioning. UCA1 downregulated miR‐128, which allowed for the overexpression of the cardioprotective protein HSP70.^48^ Further, Hao et al. found that lncRNA GAS5 decreases cardiomyocyte apoptosis by inhibiting Sema3a. Additionally, GAS5 was found to be upregulated in the myocardial infarct boundary zone in a mouse model.^49^

Myocardial syncytium disruption can lead to a number of arrhythmias. Connexin43 is one protein found in gap junctions that contributes to the syncytium of the heart. Zhang et al. found that overexpression of lncRNA CCRR in a mouse heart failure model improved cardiac conduction by preventing the degradation of connexin43 by inhibiting endocytic trafficking.^50^ Finally, Zhu et al. found that exosomes released from human umbilical cord MSCs contain MALAT1, which was shown to inhibit the NF‐κB/TNF‐α pathway in cardiac inflammation.^51^ In a similar vein, Sun et al. showed that MALAT1 regulated cardioprotective effects of myricetin in H9c2 by inhibiting the NF‐κB pathway.^52^ Key data are summarized in Table 3.

### Nervous system

Ischemic strokes are a common cause of brain injury, and promoting neuronal survival after ischemia is essential when developing a treatment. Ruan et al. found that polydactin upregulates MALAT1 and contributes to the protection of the blood brain barrier via the C/EBPβ/MALAT1/CREB/PGC‐1α/PPARγ pathway in an in vivo ischemic stroke model.^53^ From the same group, Bassit et al. found that MALAT1 associates with serine–arginine‐rich splice factor 2 to promote the alternative splicing of PKCδII. This, in turn, enhances neuronal survival through the anti‐apoptotic protein Bcl‐2.^54^ In the neonatal population, hypoxic–ischemic encephalopathy is an ischemic condition that results from compromised cerebral blood flow and causes disability. Yuan et al. found that geniposide protected PC‐12 cells against oxygen and glucose deprivation. Geniposide was found to upregulate the lncRNA H19, and the drug's protective effects were removed when H19 was silenced.^55^

**FIGURE 2:**
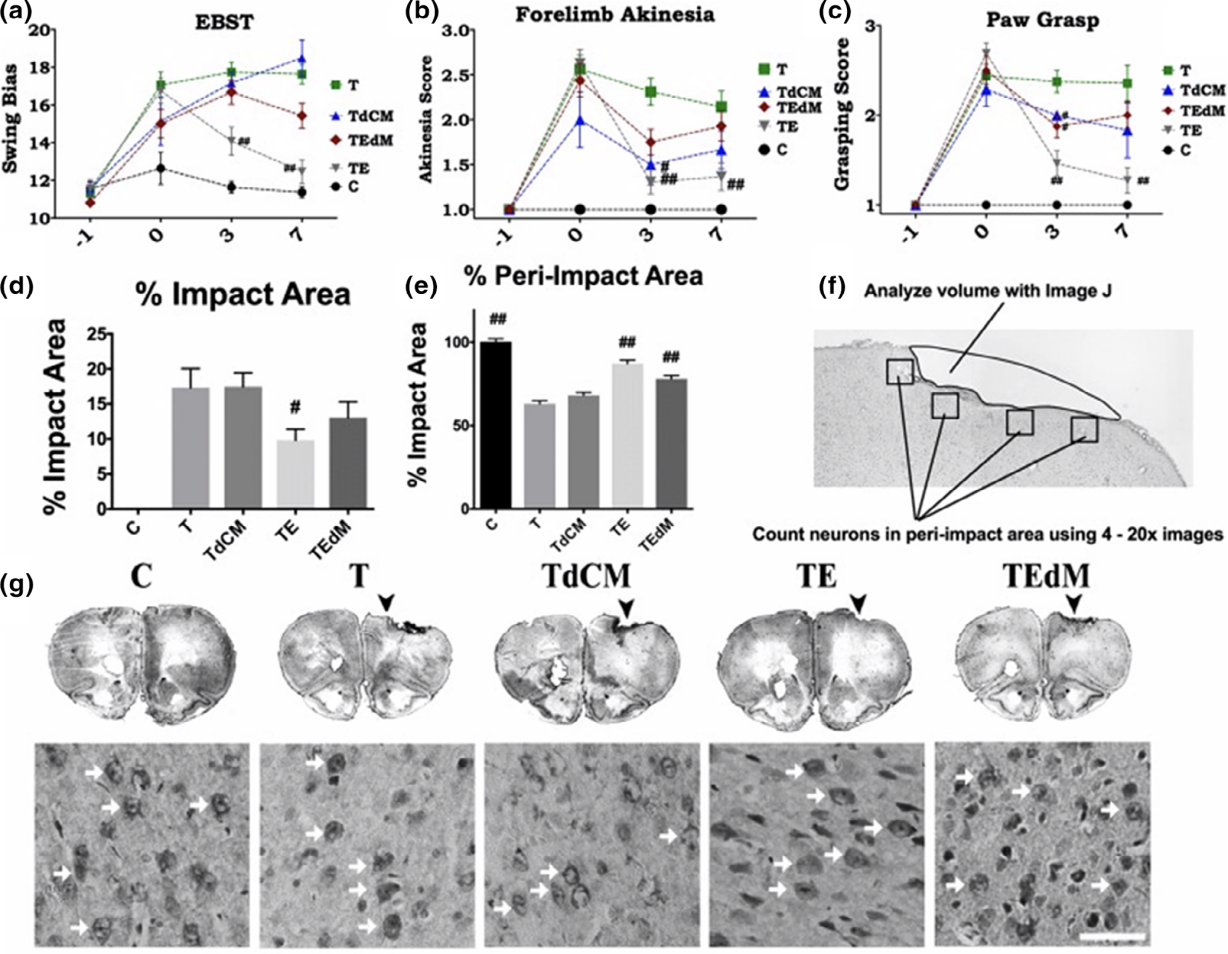
Exosomes from human adipose‐derived stem cells exhibit neuroprotective activity that is reduced upon depletion of long noncoding RNA (lncRNA) MALAT1. Graphs compare motor assessments of rats grouped as follows: surgery with no traumatic brain injury (TBI) (sham control C, *n* = 11), TBI with unconditioned media as vehicle (T; *n* = 20), TBI treated with exosomes (TE, *n* = 18), TBI treated with exosomes depleted of MALAT1 (TEdM, *n* = 20), and TBI with injection of conditioned media depleted of exosomes (TdCM; *n* = 7). Each rat was subjected to a series of behavioral tests—(a) elevated body swing test (EBST), (b) forelimb akinesia, and (c) paw grasp test—to assess motor and neurological performance of animals, at baseline before surgery and post‐TBI at Days 0, 3, and 7. Two‐way analysis of variance (ANOVA) showed significant effects as follows: EBST, treatment effects *F*(4) = 27.04; forelimb akinesia treatment effect *F*(4) = 30.3; paw grasp treatment effect *F*(4) = 42.2. Post hoc Bonferroni multiple comparisons are reported for differences versus TBI vehicle (T). #*p* < .01, ##*p* < .001. Treatment with exosomes depleted of MALAT1 (TEdM) did not improve motor performance on EBST and only improved forelimb akinesia and paw grasp at Day 3. Treatment with conditioned media depleted of exosomes (TdCM) also showed no improvement on EBST and only improved scores at Day 3 on the other two tasks. Lesion assessment: treatment with exosomes derived from hASCs significantly reduces impact and peri‐impact areas of rats after mild TBI. Nissl staining as shown in (f) was performed on Day 11 to assess damage to cortical region post TBI. Graphs (d) and (e) quantify the data from images. (f) The methods for quantifying the impact area and for choosing images for analysis of the peri‐impact area. Data for impact area (d) showed significant reduction in cortical lesion area following treatment with exosomes in the TE group and no rescue by any other treatment. Representative images of sections used for quantifying impact area and peri‐impact are shown (g). For the peri‐impact area (e), there was a significant rescue in the TE group, whereas TEdM group displayed partial rescue of the peri‐impact areas when compared with vehicle (T) and sham controls (C). Data in the bar graphs represent the mean ± *SEM* values. Impact area *F* = 14.78; peri‐impact area *F* = 56.58. Data were analyzed by one‐way analysis of variance (ANOVA) followed by Dunnett's multiple comparison test. #*p* < .1, ##*p* < .01. Data reproduced from Reference 56 (open access) with no changes

Another form of brain injury occurs by direct insult. Patel et al. found that exosomes derived from human adipose MSCs exhibited therapeutic properties when administered intravenously in a murine traumatic brain injury model. These exosomes were found to aid in recovery of motor function and reduction in cortical brain lesions via delivery of MALAT1, as exosomes depleted of MALAT1 were not efficacious (Figure 2).^56^ Key data are summarized in Table 4.

### Gastrointestinal system

Liver failure is a major threat to human health, and although liver transplant is a viable option, it is limited by cost and donor availability. Increasing the proliferation of hepatocytes is one means of a treatment. Jin et al. showed that EVs derived from human adipose MSCs reduced the amount of liver damage in an in vivo murine acute liver failure model via delivery of lncRNA H19. When H19 was silenced in MSCs, EV efficacy dropped significantly.^57^ Liver fibrosis is also associated with a high death rate, and reducing scaring of the liver is important in developing a treatment. Dong et al. found that the lncRNA GAS5 sponges miR‐23a to inhibit the PI3K p85/Akt/mTOR/Snail pathway and, in turn, restrains hepatic fibrosis in a murine model.^58^ Finally, intestinal epithelial injury is a notable hallmark of inflammatory bowel diseases and can also result from sepsis and gastrointestinal radiation. Thus, regenerating these cells would aid in the damaging effects from such conditions. Geng et al. found that lncRNA H19 induced proliferation of intestinal epithelial cells and promoted mucosal healing in an LPS‐induced inflammatory injured intestine. H19 was found to inhibit the expression of p53, miR‐34a, and let‐7, enabling cell growth and proliferation.^59^ Key data are summarized in Table 5.

### Summary of lncRNA therapeutic activity for tissue repair and regeneration

Several lncRNAs stand out for their versatile potential for regenerative applications, especially MALAT1, H19, and GAS5. The prevalence of studies reporting effects of MALAT1, in particular, is not surprising as MALAT1 is one of the most well‐characterized lncRNAs as of this writing. As its name indicates, MALAT1 (metastasis‐associated lung adenocarcinoma transcript 1) is of interest for cancer research as both a biomarker and druggable target. This dual nature is reminiscent of many miRNAs and proteins. For example, VEGF is well known as both a target of anticancer drugs (e.g., bevacizumab) as well as a candidate for therapeutic angiogenesis approaches for treatment of ischemic tissues. Thus, as lncRNA therapeutics are developed, it may be useful to observe how similarly dual‐natured molecules have been studied to determine where the boundaries between useful therapeutics and dangerous side‐effects lie.

## THERAPEUTIC ACTIVITY OF lncRNA IN CANCER

With respect to cancer, lncRNAs have mainly been viewed as potential biomarkers and as oncogenic mediators. Recently, though, there have been increasing reports of therapeutic activity of lncRNAs in cancer. In addition to their direct activity, lncRNAs have also shown potential as sensitizers for chemotherapeutic drugs. In this section, the therapeutic effects of lncRNAs relevant to the breast cancer, gastrointestinal cancer, brain and spinal cord cancer, lung cancer, skin cancer, endocrine cancer, and urinary cancer are discussed.

**TABLE 6:** lncRNA activity in breast cancer

lncRNA	Bioactivity	Reference
MEG3	Suppresses tumor growth	^61^
EGOT	Paclitaxel sensitizer	^63^

Abbreviation: lncRNA, long noncoding RNA.

**TABLE 7:** lncRNA activity in the gastrointestinal cancer

lncRNA	Bioactivity	Reference
LOC441178	Suppresses tumor growth	^64^
C5orf66‐AS1	Suppresses tumor growth	^65^
MPRL	Cisplatin sensitizer	^67^
LINC00675	Inhibits metastasis	^68^
TUBA4B	Suppresses tumor growth and inhibits metastasis	^69^
MEG3	Suppresses tumor growth	^71^
lncRNA‐APC1	Suppresses tumor growth	^72^
ENST00000547547	Suppresses tumor growth	^73^
LINC00312	Suppresses tumor growth and inhibits metastasis	^74^
MEG3	Oxaliplatin sensitizer	^77^
LINC00261	Cisplatin sensitizer	^78^
LINC00052	Suppresses tumor growth and inhibits metastasis	^79^
MIR31HG	Suppresses tumor growth	^80^
RAD51‐AS1	Etoposide sensitizer	^81^
MEG3	Inhibits epithelial‐to‐mesenchymal transition and suppresses tumor growth.	^82^, ^83^

Abbreviation: lncRNA, long noncoding RNA.

**TABLE 8:** lncRNA activity in neurological cancer

lncRNA	Bioactivity	Reference
PTCSC3	Suppresses tumor growth	^84^
GAS5	Cisplatin sensitizer	^85^
AC003092.1	Temozolomide sensitizer	^86^

Abbreviation: lncRNA, long noncoding RNA.

**TABLE 9:** lncRNA activity in lung cancer

lncRNA	Bioactivity	Reference
LOC285194	Suppresses tumor growth	^88^
FENDRR	Suppresses tumor growth	^89^
STXBP5‐AS1	Suppresses tumor growth	^90^
MEG3	Potential effector of paclitaxel	^91^

Abbreviation: lncRNA, long noncoding RNA.

**TABLE 10:** lncRNA activity in bone cancer

lncRNA	Bioactivity	Reference
SRA1	Suppresses tumor growth	^92^
CTA	Doxorubicin sensitizer	^94^

Abbreviation: lncRNA, long noncoding RNA.

**TABLE 11:** lncRNA activity in skin cancer

lncRNA	Bioactivity	Reference
LINC00520	Suppresses tumor growth	^95^
MEG3	Cisplatin and 5‐FU sensitizer	^96^
GAS5	Suppresses tumor growth	^97^

Abbreviation: lncRNA, long noncoding RNA.

**TABLE 12:** lncRNA activity in endocrine cancer

lncRNA	Bioactivity	Reference
LINC01186	Suppresses tumor growth	^98^
NEN885	Inhibits epithelial‐to‐mesenchymal transition	^100^
MEG3	Suppresses tumor growth	^101^

Abbreviation: lncRNA, long noncoding RNA.

**TABLE 13:** lncRNA activity in urinary cancer

lncRNA	Bioactivity	Reference
GAS5	Sorafenib sensitizer	^102^
PTENP1	Suppresses tumor growth	^103^
LOC572558	Suppresses tumor growth	^104^

Abbreviation: lncRNA, long noncoding RNA.

### Breast cancer

Breast cancer is the second most common type of cancer in women in the United States.^60^ Zhang et al. showed that overexpression of lncRNA MEG3 in breast cancer cells acted as a tumor suppressor in vitro and in an in vivo xenograft model. This was found to be due in part to MEG3 enhancing ER‐stress‐related genes and inducing NF‐kB and p53 signaling pathways.^61^ Another lncRNA, NKILA, was shown to activate similar pathways.^62^ The sensitivity of breast cancer cells to paclitaxel was enhanced by the antisense intronic lncRNA EGOT due to the upregulation ITPR1, as reported by Xu et al.^63^ Key data are summarized in Table 6.

### Gastrointestinal cancer

Oropharyngeal cancer affects the region of the head and neck. Xu et al. found that lncRNA LOC441178 suppressed the invasion and migration of squamous carcinoma cells (SCCs) via targeting rho‐associated, coiled‐coil‐containing protein kinase 1.^64^ Similarly, Lu et al. reported that lncRNA C5orf66‐AS1 regulated the expression of cytochrome c1 and inhibited the proliferation, migration, and invasion of oral SCCs.^65^ Furthermore, Zhao et al. showed that lncRNA RP11‐169D4.1 sequestered miR‐205‐5p and regulated the expression of CDH1 in laryngeal squamous cell carcinoma.^66^ A study of squamous cell carcinoma of the tongue by Tian et al. found that lncRNA MPRL controlled cisplatin sensitivity by directly binding to pre‐miR‐483 and preventing its maturation to miR‐483‐5p thereby allowing for upregulation of its target FIS1.^67^

Immediately after the oropharynx in the gastrointestinal tract is the esophagus. Zhong et al. found that lncRNA LINC00675 acted as an anti‐metastatic factor in esophageal squamous cell carcinoma by inhibiting the Wnt/β‐catenin pathway.^68^ The esophagus culminates in the stomach, which is affected by gastric cancer. Guo et al. found that overexpressing lncRNA TUBA4B inhibited gastric cancer cell proliferation and invasion as well as induced apoptosis in vitro. TUBA4B overexpression also prevented tumor growth and metastasis in vivo, with a potential mechanism of sponging miR‐214 and miR‐216a/b to increase levels of PTEN and inhibit the PI3K/AKT signaling pathway.^69^ Similarly, Cen et al. found that the PI3K/AKT signaling pathway was inhibited when lncRNA STXBP5‐AS1 was overexpressed in these same cells.^70^ Also, Peng et al. showed that lncRNA MEG3 was able to sponge the miR‐181 family and subsequently upregulate Bcl‐2 to suppress gastric cancer.^71^

Cancers of the large intestine are referred to as colorectal cancers, denoted by their formation in part of the colon or the rectum. Wang et al. found that silencing lncRNA‐APC1 in colorectal carcinoma cells (CRCs) caused increased levels of Wnt1 in their exosomes, resulting in increased proliferation and migration of the CRCs.^72^ Another study by Ai et al. found that lncRNA ENST00000547547 acted as a tumor suppressor in colorectal cancer by inhibiting the proliferation, migration, and invasion of CRCs leading to decreased tumorigenesis in vivo.^73^ In addition, Li et al. demonstrated that lncRNA LINC00312 decreased CRC proliferation, migration, and invasion in vitro as well as attenuated tumor development and metastasis in vivo utilizing multiple CRC lines. They identified the ability of LINC00312 to sponge miR‐21 and regulate the miR‐21/PTEN axis.^74^ Dai et al. described the anti‐apoptotic effects of berberine in CRCs. Berberine upregulated lncRNA CASC2, which bound AUF1 resulting in inhibition of Bcl‐2 translation.^75^ Chang et al. showed that the extract EGb 761 from the *Ginkgo biloba* plant increased levels of lncRNA lincRNA‐p21. The lncRNA was found to prevent ubiquitination of E‐cadherin, thereby stabilizing cell–cell junctions and mediating anti‐colon cancer effects.^76^ Finally, related to drug sensitization, one study found that upon overexpression of lncRNA MEG3 in oxaliplatin‐resistant cells lines, MEG3 sequestered miR‐141 and led to the upregulation of PDCD4, sensitizing HCT116/OXA cells to oxaliplatin.^77^ A separate study found that overexpression of lncRNA LINC00261 reduced cisplatin resistance in a mouse model of colon cancer.^78^

While not directly part of the gastrointestinal tract, the liver and gall bladder play critical roles in gastrointestinal function. Studying hepatocellular carcinoma, Yan et al. found that lncRNA LINC00052 upregulated the expression of miR‐101‐3p and consequently decreased hepatocellular carcinoma cell (HCC) proliferation and metastasis.^79^ Yan et al. showed that lncRNA MIR31HG suppressed hepatocellular carcinoma by sponging miR‐575 to inhibit ST7L expression.^80^ Another study by Chen et al. found that melatonin was able to interfere with DNA repair mechanisms by inhibiting the translation of RAD51. Specifically, melatonin induced expression of lncRNA RAD51‐AS1 that was able to bind to RAD51 mRNA to prevent the production of RAD51 protein. This led to increased sensitivity of the chemotherapeutic etoposide in a xenograft mouse model using HCCs.^81^ Fan et al. showed that the epithelial‐to‐mesenchymal transition of HCCs was inhibited by treatment with arsenic trioxide via upregulation of lncRNA MEG3, which in turn, downregulated PKM2.^82^ Demonstrating the multitude of pathways that can be affected by a single lncRNA, Jin et al. found that MEG3 promoted the degradation of the histone methyltransferase protein EZH2, which allowed for increased expression of the tumor suppressor protein LATS2 in gallbladder cancer cells.^83^ Key data are summarized in Table 7.

### Neurological cancer

Tumors that develop in the brain and spinal cord are classified as gliomas. Xia et al. found that lncRNA PTCSC3 suppressed proliferation, migration, and invasion of glioma cell lines, and further found that these functional effects were due to PTCSC3's downregulation of LRP6 and subsequent suppression of the Wnt/βcatenin signaling pathway.^84^ Huo et al. found that lncRNA GAS5 was downregulated in glioma cell lines with low sensitivity to cisplatin. By overexpressing GAS5, they found that glioma cells were sensitized to cisplatin by restoring cisplatin‐inhibited mammalian target of rapamycin activation.^85^ A Stage IV glioma is classified as a glioblastoma and is the most aggressive type of cancer that develops in the brain. Xu et al. found that lncRNA AC003092.1 was decreased in glioblastoma cells resistant to temozolomide. When the lncRNA was overexpressed, sensitivity of glioblastoma cell lines to temozolomide was enhanced resulting in increased apoptosis through a TFPI‐2 mediated pathway.^86^ Key data are summarized in Table 8.

### Lung cancer

Lung cancer is the leading cause of cancer‐related deaths in both men and women.^87^ Zhou et al. found that lncRNA LOC285194 acted as a tumor suppressor in non‐small cell lung cancer by regulating p53 and suggested that the lncRNA is involved in regulating the KRAS/BRAF/SMEK pathway.^88^ Zhang et al. showed that lncRNA FENDRR inhibited the proliferation, migration, and invasion capacities of non‐small cell lung cancer in in vitro and in vivo studies.^89^ In addition, Huang et al. demonstrated that lncRNA STXBP5‐AS1 acts as a tumor suppressor in non‐small cell lung cancer by inhibiting cell proliferation, migration, and invasion.^90^ Further, Xu et al. found that paclitaxel for advanced non‐small cell lung cancer decreased proliferation of such cells by upregulating lncRNA MEG3, which increased the expression of p53.^91^ Key data are summarized in Table 9.

### Bone cancer

Osteosarcoma is the most common form of bone cancer. Guo et al. found that lncRNA SRA1 was able to inhibit proliferation, migration, and invasion as well as facilitate apoptosis by sponging miR‐208a in osteosarcoma cells.^92^ Another study found that lncRNA FER1L4 sequestered miR‐18a‐5p in order to modulate the expression of PTEN in osteosarcoma cells.^93^ Additionally, Wang et al. showed that lncRNA CTA was downregulated in doxorubicin‐resistant osteosarcoma cells. When CTA was overexpressed, doxorubicin resistance was overcome in vitro and in vivo. CTA was found to promote apoptosis of osteosarcoma cells via sponging miR‐210.^94^ Key data are summarized in Table 10.

### Skin cancer

There are three types of cancers that can develop in the skin. Basal cell carcinoma and squamous cell carcinoma arise from keratinocytes, while melanoma arises from melanocytes. Mei et al. found that lncRNA LINC00520 targeted EGFR inhibition and resulted in the inactivation of the PI3K/Akt pathway leading to inhibition of cutaneous squamous cell carcinoma development.^95^ Long et al. found that lncRNA MEG3 regulated the levels of E‐cadherin, N‐cadherin, and cyclin D1 in melanoma cells through CYLD expression by sponging miR‐499‐5p. In the same study, MEG3 was also found to increase the sensitivity of melanoma cells to cisplatin and 5‐FU treatment.^96^ Also, Chen et al. reported that lncRNA GAS5 played an anticancer role in melanoma via regulating Gelatinase A and B.^97^ Key data are summarized in Table 11.

### Endocrine cancer

**FIGURE 3:**
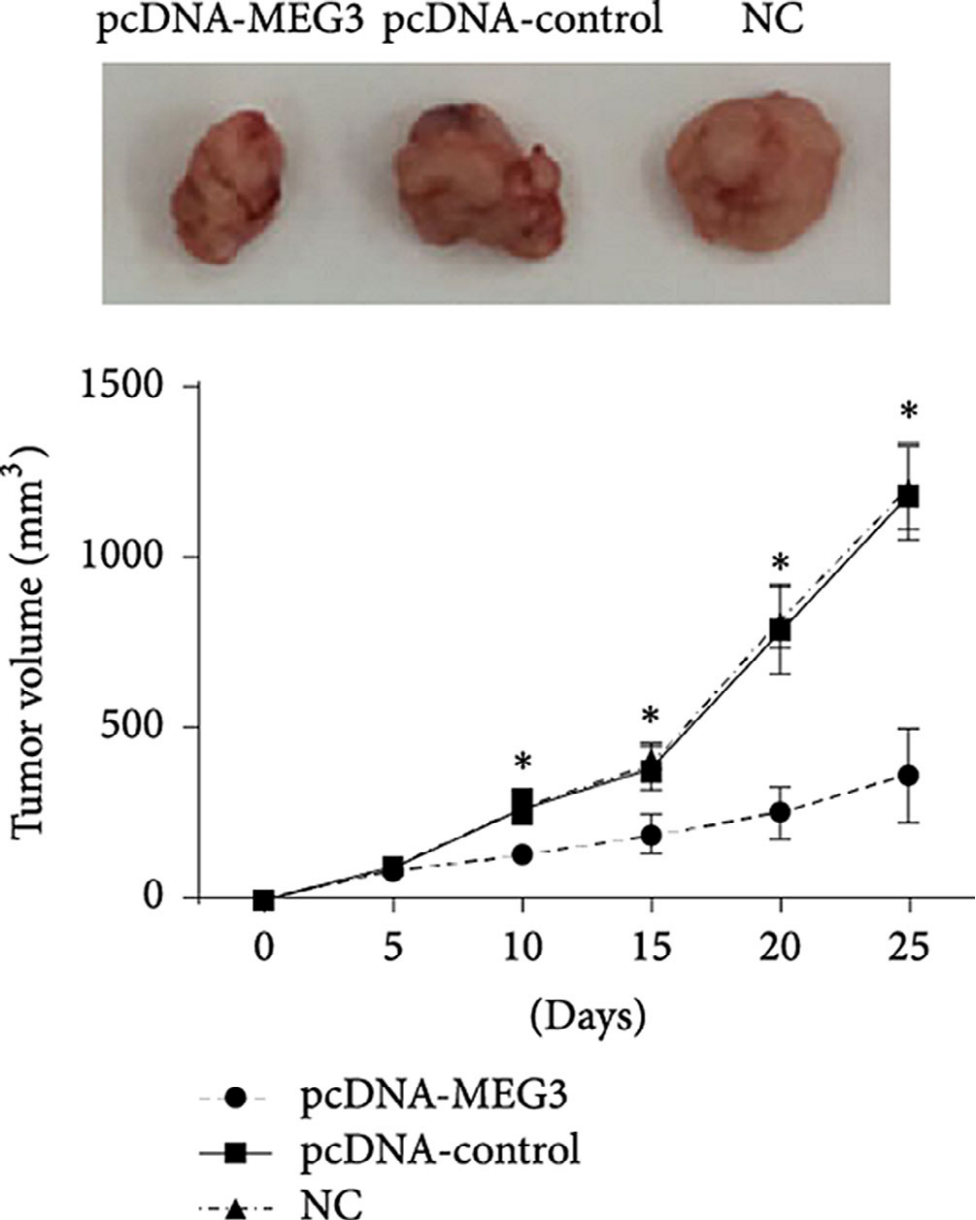
MEG3 overexpression impedes melanoma growth. A375 cells were transplanted subcutaneously into nude mice (25–30 g, 6 weeks old, *n* = 6 per group) after either no transfection (NC), transfection with a plasmid to overexpress MEG3 (pcDNA‐MEG3), or transfection with the control plasmid (pcDNA control). Upregulation of MEG3 decreased the tumor volume and weight (*p < .05). Data reproduced from Reference 101 (open access) without changes

Working in thyroid cancer, the most common type of endocrine cancer, Wang et al. found that lncRNA LINC01186 overexpression decreased cell proliferation, colony formation, and invasion in papillary thyroid carcinoma cell lines TPC‐1 and IHH‐4.^98^ Cancers of the pancreas can develop both exocrine and endocrine tumors.^99^ Wei et al. found that lncRNA NEN885 regulated cellular migration and invasion in gastroenteropancreatic neuroendocrine neoplasms by activating the epithelial‐to‐mesenchymal transition partially through canonical Wnt/β‐catenin signaling.^100^ Also, Gu et al. found that overexpression of lncRNA MEG3 was able to suppress pancreatic cancer activity by regulating PI3K/AKT/Bcl‐2/Bax/Cyclin D1/P53 and PI3K/AKT/MMP‐2/MMP‐9 signaling pathways (Figure 3).^101^ Key data are summarized in Table 12.

### Urinary cancer

Urinary cancers include malignancies of both the kidneys and the bladder. Liu et al. found that lncRNA GAS5 was decreased in sorafenib‐resistant renal cell carcinoma cells, but overexpression of GAS5 sensitized the cells to the drug by sequestering miR‐21 and upregulating SOX5.^102^ In the bladder, Zheng et al. showed that normal bladder cells released exosomes that contained the lncRNA PTENP1. This lncRNA was shown to stabilize the tumor suppressor gene PTEN by sequestering miR‐17, increasing bladder cancer cell apoptosis and decreasing migratory properties in vitro as well as decreasing tumor growth in an in vivo model.^103^ Further, Zhu et al. found that lncRNA LOC572558 acted as a tumor suppressor in bladder cancer. Overexpression of this lncRNA decreased proliferation and increased apoptosis of bladder cancer cells in vitro.^104^ Key data are summarized in Table 13.

### Summary of therapeutic activity of lncRNA in cancer

The therapeutic potential of lncRNAs in cancer is still a nascent area of study, as lncRNAs are most commonly studied as possible cancer biomarkers or drug targets. These studies reveal a confluence of signaling pathways regulated by lncRNAs in cancer, and not surprisingly many of the most studied pathways are listed (e.g., PI3K, KRAS). As more dedicated studies of lncRNA activity in cancer are conducted, it is likely that a greater diversity of pathway interactions will be reported. It is also notable that MEG3 appears to be the most versatile lncRNA with regard to therapeutic potential in cancer, and thus this lncRNA may be a good candidate for future EV delivery studies.

## EV DELIVERY OF lncRNA

**FIGURE 4:**
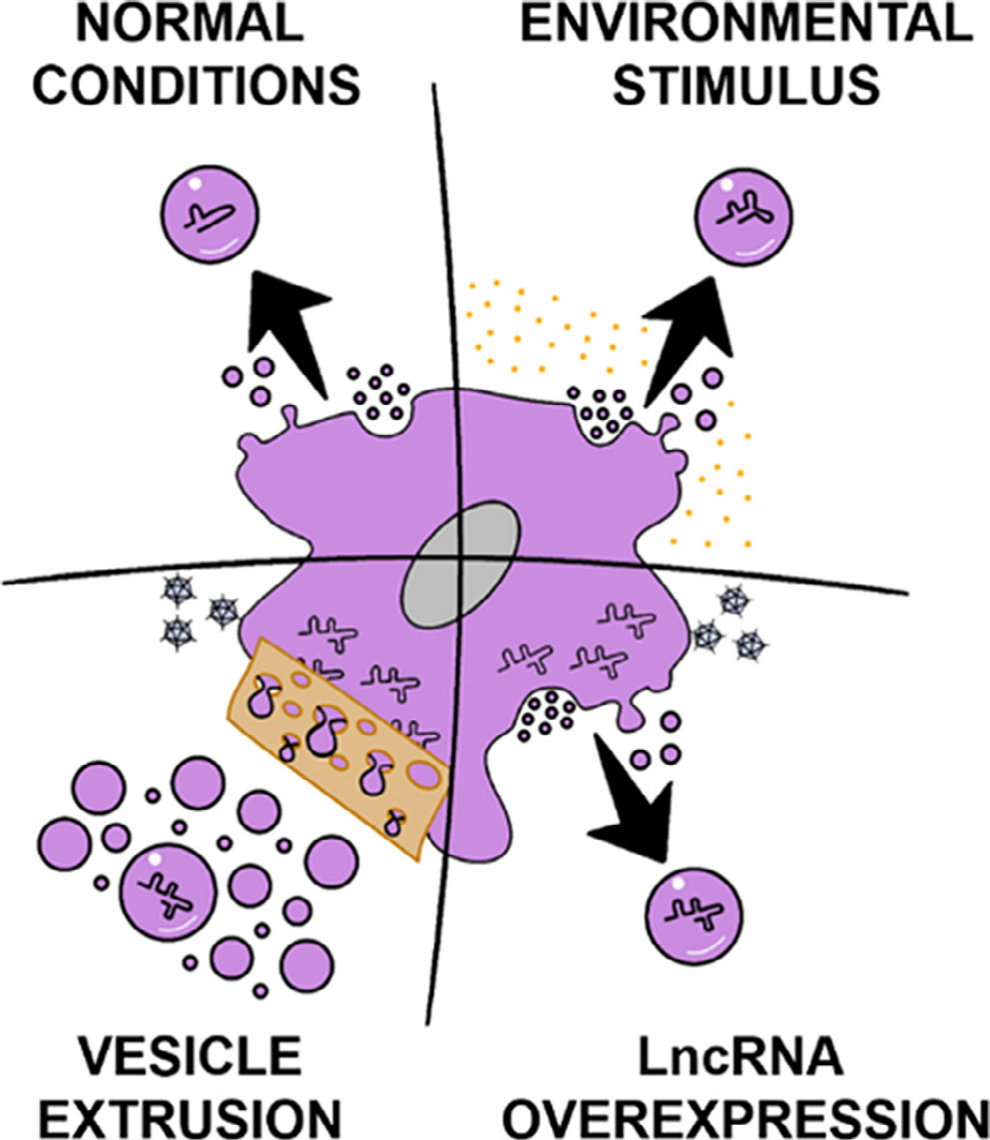
Methods to control long noncoding RNA (lncRNA) loading into extracellular vesicles (EVs). Top left, EVs could be isolated from cells known to release vesicles enriched in an lncRNA of interest. Top right, cells could be cultured in an environment that causes enrichment of a specific EV‐associated lncRNA. Bottom right, lncRNAs of interest could be overexpressed via cellular transfection/transduction resulting in stoichiometric enrichment in EVs. Bottom left, lncRNAs of interest could be overexpressed via cellular transfection/transduction followed by creation of EV‐mimics, for example, by filter extrusion

As with other RNA therapeutics, there is a presumed need to protect therapeutic lncRNAs from circulating nucleases to enable efficacy. As synthetic lncRNAs are currently not widely available, utilization of EVs as natural lncRNA delivery vehicles is of high interest. Within this paradigm, several approaches have been taken (Figure 4). One method is collecting exosomes from a cell type known to secrete EVs enriched with an lncRNA of interest. (Figure 4, top left). Although there have been relatively few studies as of this writing, this method has been the most popular. Adult stem/stromal cells^32^, ^33^, ^56^, ^57^ and progenitor cells^43^ are common choices due to their inherent regenerative properties. This approach is advantaged by its straightforward nature; however, potency of unmodified EVs may be limited, resulting in the need for high EV doses that could induce undesired effects.

A second approach is to control the environmental conditions of cells to specifically tailor EV content. This strategy relies on the ideas that cell phenotype is responsive to stimuli and that cell phenotype dictates EV cargo (Figure 4, top right). Some studies suggest that RNA sequence motifs are responsible for loading lncRNA into EVs.^105^, ^106^ A particular subset of EVs released in response to a stimulus may be preferentially loaded based on interactions with newly expressed EV‐loading proteins that are able to interact with these motifs. Thus, an lncRNA‐EV‐mediated response to an external stressor may not directly rely on cellular levels of lncRNAs, but rather on the expression of EV cargo‐loading machinery. One study focusing on exosomal lncRNA content after DNA damage found that it did not correlate with cellular lncRNA content.^107^ While loading EVs via environmental cues is still limited by our knowledge of cellular responses to stimuli in culture, it is useful when combined with well‐characterized phenomena. For example, both ethanol preconditioning^11^ and hyperbaric oxygen stimulation^10^ have been shown to regulate EV lncRNA content.

Additionally, EVs have also been loaded with lncRNA by manipulation of cellular lncRNA content. Overexpression of cellular lncRNA stoichiometrically favors loading of EVs with that particular lncRNA (Figure 4, bottom right), as demonstrated by Jin et al.^57^ This approach has been also been modified for the creation of EV‐mimetics via extrusion of lncRNA‐overexpressing cells (Figure 4, bottom left). Creation of mimetics in this manner results in high vesicle yields,^108^ although it is unclear if the favorable physiological properties of EVs would be retained via this approach, and reproducibility and scalability also present challenges.

Exogenous loading techniques have also been developed for EVs and could be applied to lncRNA loading once synthetic production of these RNAs is achieved. A common technique for loading EVs with nucleic acids is electroporation, as seminally reported by Wood et al.^109^ More recently, Kao et al. utilized electroporation to load large (~7 kb) DNA plasmids into human megakaryocytic microparticles at thousands of copies per particle.^110^ This result suggests that some level of lncRNA loading may be achievable using this process. Beyond electroporation, sonoporation has also been used successfully for EV loading of both nucleic acids^111^ and large proteins such as catalase,^112^ a ~240 kDa enzyme. Additional methods, such as the use of pH‐gradient‐mediated loading^113^ or cellular nanoporation,^114^ among others, may also eventually be useful for loading synthetic lncRNAs into EVs.

## FUTURE CONSIDERATIONS

In addition to the need for better mechanistic understanding of lncRNA activity, there are several open questions whose answers will be critical in defining appropriate dose and scheduling considerations for EV delivery of lncRNA. For example, it is not clear how many copies of a particular lncRNA per cell are needed to achieve an effect or how long lncRNA effects last. This issue is especially critical as the relatively large size of lncRNAs should limit their loading capacity in EVs (or any other delivery vehicle) relative to other ncRNAs such as small interfering RNA and miRNA. Identifying off‐target effects of lncRNAs, especially for therapies requiring systemic administration, is also necessary to ensure safety. To this end, knowledge about potential toxicity of lncRNAs, both intracellularly and extracellularly, must be established. Further, while EV‐associated lncRNAs are thought to act primarily in the cytoplasm as miRNA sponges, they could perform other functions, such as serving as scaffolds for transcriptional complexes following transport to the nucleus. Understanding these phenomena is paramount to developing effective EV‐lncRNA therapeutics.

## CONCLUSIONS

lncRNAs are an exciting class of regulatory RNAs with increasing rates of newly discovered therapeutic properties. They are naturally contained within EVs in the body, promoting optimism for the development of a novel class of EV‐based therapies. However, a number of hurdles must be overcome for EV‐lncRNA‐based treatments to be realized, including improved understanding of mechanisms of action, pharmacokinetics, and toxicity. Addressing these issues, combined with further developments in generation of synthetic lncRNAs and improved biomanufacturing of EVs, will be required to enable the full potential of EV‐lncRNA therapeutics.

## CONFLICT OF INTERESTS

The authors declare no conflict of interest.
